# Synthesis of Curcumin Derivatives and Analysis of Their Antitumor Effects in Triple Negative Breast Cancer (TNBC) Cell Lines

**DOI:** 10.3390/ph12040161

**Published:** 2019-10-26

**Authors:** Paola Maria Bonaccorsi, Manuela Labbozzetta, Anna Barattucci, Tania Maria Grazia Salerno, Paola Poma, Monica Notarbartolo

**Affiliations:** 1 Department of Chemical, Biological, Pharmaceutical and Environmental Science, University of Messina, 98122 Messina, Italy; pbonaccorsi@unime.it (P.M.B.); abarattucci@unime.it (A.B.); tsalerno@unime.it (T.M.G.S.); 2 Department of Biological, Chemical and Pharmaceutical Science and Technology (STEBICEF), University of Palermo, 90133 Palermo, Italy; manuela.labbozzetta@unipa.it (M.L.); monica.notarbartolo@unipa.it (M.N.)

**Keywords:** antiproliferative activity, prooxidant activity, antioxidant activity, pro-apoptotic activity, NF-κB inhibition, sulfenic acid

## Abstract

We analyzed antitumor effects of a series of curcumin analogues. Some of them were obtained by reaction of substitution involving the two phenolic OH groups of curcumin while the analogues with a substituent at C-4 was prepared following an original procedure that regards the condensation of benzenesulfenic acid onto the nucleophilic central carbon of the curcumin skeleton. We analyzed cytotoxic effects of such derivatives on two TNBC (triple negative breast cancer) cell lines, SUM 149 and MDA-MB-231, but only three of them showed an IC_50_ in a lower micromolar range with respect to curcumin. We also focused on these three derivatives that in both cell lines exhibited a higher or at least equivalent pro-apoptotic effect than curcumin. The analysis of molecular mechanisms of action of the curcumin derivatives under study has highlighted that they decreased NF-κB transcriptional factor activity, and consequently the expression of some NF-κB targets. Our data confirmed once again that curcumin may represent a very good lead compound to design analogues with higher antitumor capacities and able to overcome drug resistance with respect to conventional ones, even in tumors difficult to treat as TNBC.

## 1. Introduction

Cancer can be described as uncontrolled DNA replication and cell division, evasion from programmed cell death, breaking through normal tissue boundaries and invasion to new sites in the body. Actually, the most promising anti-cancer therapies combine agents with different molecular mechanisms such as specific drugs and chemo- or radiotherapies, resulting in a better efficacy and longer survival [1]. 

In this scenario, polyphenols are to be considered molecules with antitumor action because of their capacity to interfere with a large number of pathways that in the neoplastic cell are simultaneously deregulated. Their role in improving bioavailability of drugs has been often underlined, perfecting the response of cancer cell to different therapies [2]. Recently, researchers suggested that natural polyphenols might be also used to sensitize tumor cells to chemo- and radiotherapy by inhibiting pathways that lead to treatment resistance [3]. For this reason, these compounds are often defined as “privileged” structures [1], the implications of which in human health are vast, including many antitumor activities, neuroprotection, beneficial effects in cardiovascular dysfunctions, diabetes and inflammatory processes. 

Curcumin, extracted from rhizomes of *Curcuma longa* L. and used for thousands of years in traditional eastern medicine, is certainly one of the most studied natural polyphenols, despite its poor bioavailability, rapid in vivo metabolism and low cellular uptake that limit their use in therapy [4]. Curcumin is still explored for its antioxidant and anti-inflammatory properties and especially for its antitumor properties [5,6,7,8] exerted towards many types of cancers, such as breast cancer, hepatocellular cancer, multiple melanoma, osteosarcoma, hematological malignances, lung cancer, head and neck squamous cell carcinoma, prostate cancer and brain tumors [9,10,11,12,13,14,15,16,17,18,19,20,21,22]. Research on curcumin over the years focused on the development of derivatives or delivery systems that could bypass the critical issues of such polyphenols and emphasize its potential health benefits [23,24,25,26,27,28,29,30,31,32,33]. 

In this context, we recognized the interest in the synthesis of curcumin derivatives that could act against a targeted typology of cancer. Therefore, we describe synthetic procedures of some curcumin derivatives and their antitumor capacities in two triple negative breast cancer (TNBC) cellular models, MDA-MB-231 and SUM 149, in comparison with curcumin. We have chosen to study these molecules on TNBC cell line models because TNBC represents one of the most aggressive malignant neoplasms, characterized by molecular aspects as the lack of the respective receptor targets, that limit therapeutic possibility because it does not respond to conventional hormonal interventions. Moreover, another peculiar characteristic is the over-expression and hyperactivation of the transcription factor NF-κB, of which curcumin is an inhibitor [34,35]. 

## 2. Results and Discussion

### 2.1. Chemistry

**Figure 1:**
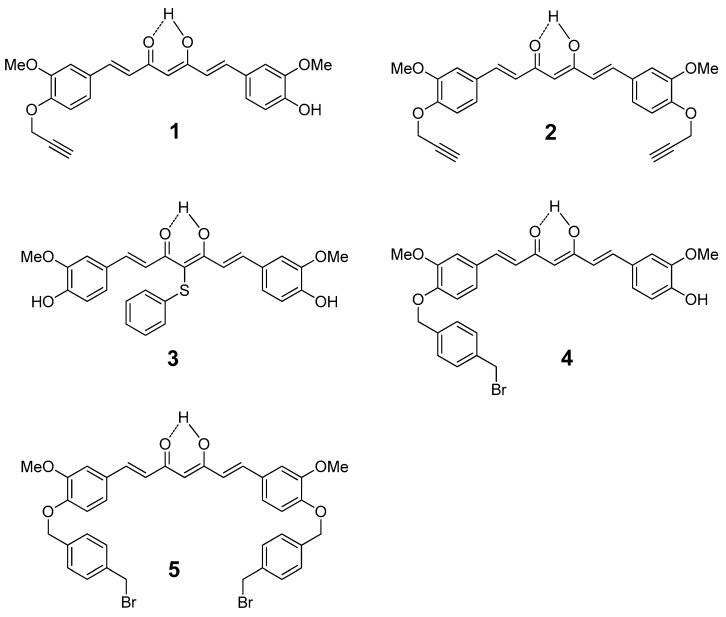
Structural skeletons of curcumin analogues **1**–**5**.

**Scheme 1:**
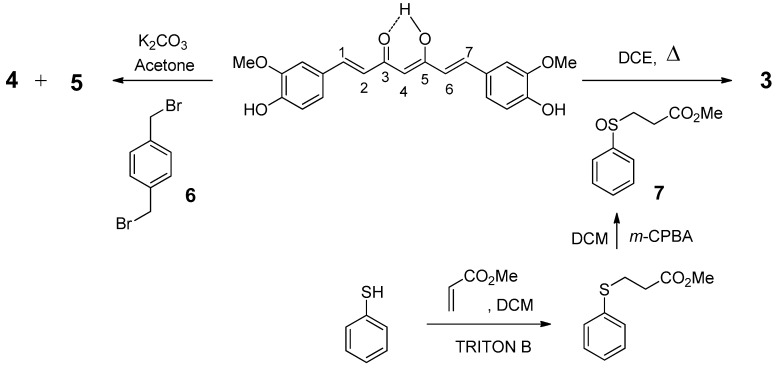
Synthetic procedures for curcumin analogues **3**–**5**.

Extensive structure-activity studies have shown that the phenolic OHs and the α,β-unsaturated di-keto groups are essential for the antioxidant properties exerted by curcumin and the α,β-unsaturated di-keto moiety, in particular, is a key for its anticancer activity. On the other hand, it is believed that the poor bioavailability and stability of curcumin in physiological media depend on these groups [36,37]. Taking into account such observations, we have chosen to synthesize and study curcumin derivatives where the phenolic OH groups are completely or partially substituted, and a curcumin derivative, the unsaturated di-keto chain of which has been substituted on C-4. Five curcumin derivatives were chosen to carry out this investigation and their structures are shown in Figure 1. Compounds **1** and **2** were known in the literature, and their synthesis (^1^H and ^13^C NMR spectra in Materials and Methods) was conducted by modification of an already described procedure [38]. Compounds **4** and **5** were obtained from curcumin and dibromo-*p*-xylene 6 in the presence of K_2_CO_3,_ separated and purified by column chromatography (Scheme 1). The substitution reaction of the phenolic function of curcumin on the di-bromo derivative 6 was complicated by the formation of an insoluble precipitate that was nearly avoided by the addition of *p*-xylene derivative 6 and a base in two tranches. Finally, Compound **3** (^1^H and ^13^C NMR spectra in Materials and Methods) was synthesized, taking into account the highly selective reaction reported by Allison [39] (Scheme 1) in which the electrophilic sulfenic function reacts with the nucleophilic central carbono f a 1,3-diketone, producing a thioether with loss of water. The mechanism of the reaction is believed to be concerted. Sulfenic acids are usually transient intermediates with a dual electrophilic/nucleophilic nature and for this reason they are generated in situ from suitable precursors [40,41]. In Scheme 1 the thermolysis of sulfoxide 7, the precursor of the corresponding sulfenic acid, is shown, together with its formation from thiophenol. The reaction was carried out in 1,2-dichloroethane (DCE) at reflux, without the presence of any base or acid [42], so avoiding any possible decomposition of the labile curcumin. 

### 2.2. Biological Studies 

#### 2.2.1. Antiproliferative Activity

**Figure 2:**
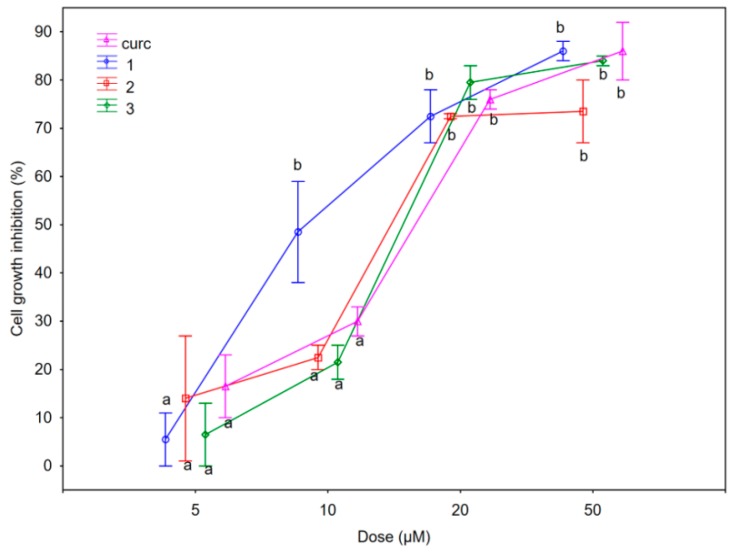
Cytotoxic activity of curcumin and its derivatives **1**–**3** on SUM 149 cells. Cell viability was assessed by MTS assay. Data are expressed as the mean of at least three different experiments performed in triplicate. Different letters represent significant differences in cytotoxic activity among the concentration (Tukey test, *p* < 0.05).

**Figure 3:**
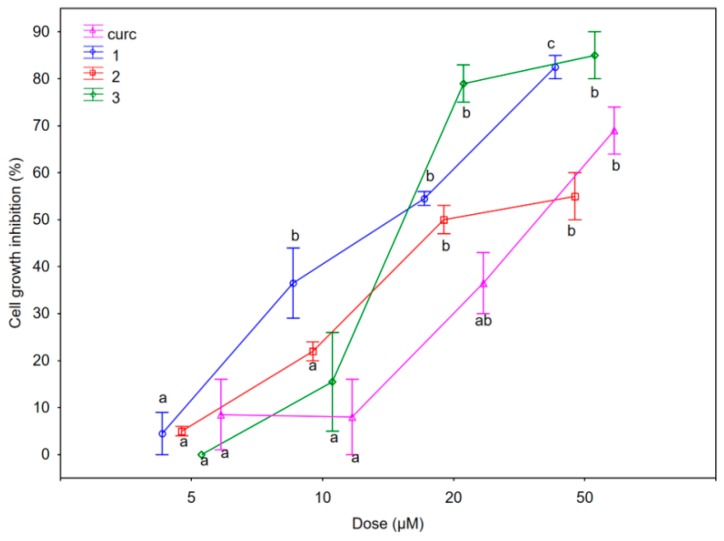
Cytotoxic activity of curcumin and its derivatives **1**–**3** on MDA-MB-231 cells. Cell viability was assessed by MTS assay. Data are expressed as mean of at least three different experiments performed in triplicate. Different letters represent significant differences in cytotoxic activity among the concentration (Tukey test, *p* < 0.05).

We analyzed cytotoxic effects of curcumin derivatives 1–5 on the two TNBC cell lines SUM 149 and MDA-MB-231, and also in a normal cell line, 1-7HB2. In Table 1 the IC_50_ of Compounds **1**–**5** and curcumin are reported and only Compounds **1**–**3** showed an IC_50_ lower than curcumin (Figure 2 and Figure 3), with SUM 149 cells being the most sensitive to the action of these derivatives. This result led us to focus our studies on Compounds **1**–**3**.

In order to verify if these analogues are characterized by a specificity of action towards the cancer model examined, we performed a cell growth assay in non-TNBC cells, in particular, in a cell line of acute promyelocytic leukemia HL60 and in its multidrug resistant variant (HL60: curcumin IC_50_ = 47.5 μM ± 3.3, 1 IC_50_ = 14.0 μM ± 1.7, 2 IC_50_ = 17.0 μM ± 1.2, 3 IC_50_ < 1 μM; HL60 R: curcumin IC_50_ = 40.5 μM ± 2.1, 1 IC_50_ = 7.0 μM ± 0.8, 2 IC_50_ = 9.0 μM ± 2.8, 3 IC_50_ = 6.7 μM ± 1.1). These results confirmed that Compounds **1**–**3** do not have specific effects on TNBC cell lines, on the contrary, they show a strong action also in the non-TNBC cancer model analyzed and for this are even more interesting, because they can be used in different types of cancer. In line with the results obtained in the TNBC cells, also in the non-TNBC cells, Analogues **1**–**3** are more active than curcumin. Noteworthy the IC_50_ of Compounds **1** and **2** are lower in the multidrug resistant variant, HL60R, than the parental HL60; these results are worthy to further experimental analyses to verify if these analogues are capable to bypass the wide problem of acquired multidrug resistance.

#### 2.2.2. Pro- and Antioxidant Activity

We investigated the mechanism by which compounds could exert any anticancer activity with the study of their pro- or antioxidant properties, due to the well-known role that oxidative stress plays in the progression of a number of cancers. Therefore, our investigation on the mechanism by which Compounds **1**–**3** could exert any anticancer activity started with the study of their pro- or antioxidant properties. 

In order to assess whether Analogues **1**–**3** could behave as pro-oxidant substances, the two TNBC cells were treated with the antioxidant N-acetyl-L-cysteine (NAC), which is a well-known scavenger of reactive oxygen intermediates, at 2mM for 1h, before exposure to curcumin and Compounds **1**–**3** at the corresponding IC_50_ values. As shown in Table 2, both for SUM 149 cells (A) and MDA-MB-231 cells (B), the addition of NAC reduced the cytotoxic activity of Compounds **1** and **2** as well as of curcumin, whereas the same results were not obtained for Compound **3**. In our study, the analysis carried out with DPPH (2,2-diphenyl-1-picrylhydrazyl) reduction assay indicated that Analogues **1** and **2** do not possess antioxidant activity, since the efficient dose (ED_50_) of these was not identified (Table 3). From a chemical point-of-view, these results are in line with previous observations showing that the protection of the phenolic groups causes the complete abolition of the antioxidant activity [43]. From a biological point of view, they indicated that the mechanism of antitumor activity against TNBC can involve an at least partially pro-oxidant effect for Compounds **1** and **2**. Surprisingly, substitution in the active methylene site of Compound **3** showed a scavenging activity lower then curcumin [43], reaching an ED_50_ at a concentration of 19.2 µM (Table 3). 

#### 2.2.3. Pro-Apoptotic Activity 

**Figure 4:**
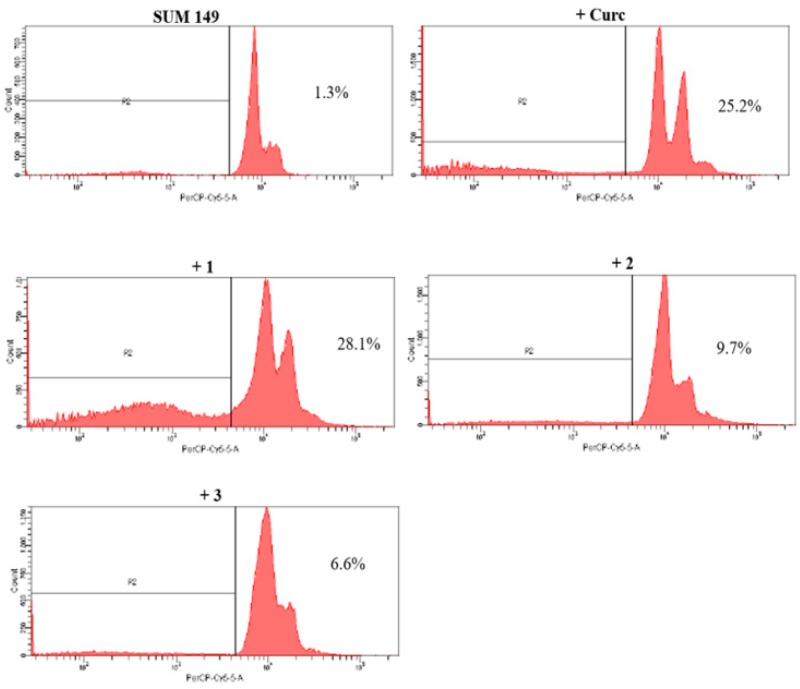
Representative example of flow cytometry analysis with propidium iodide. SUM 149 cells were treated with curcumin and its analogues at 15 μM for 24 h. Numbers in the panels indicate the % of the events in the preG_0_–G_1_ position.

**Figure 5:**
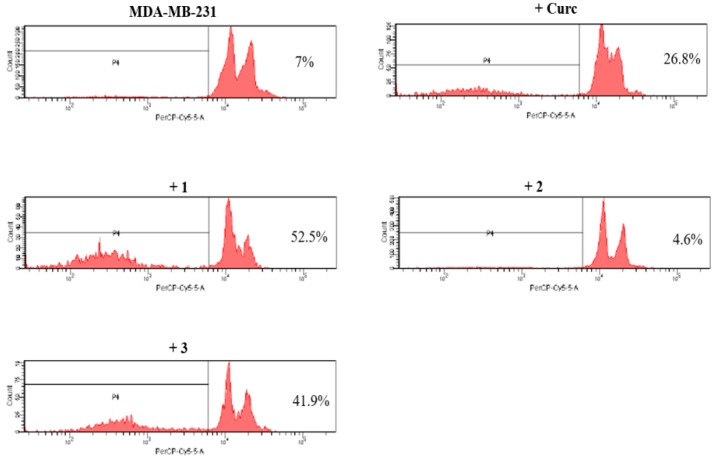
Representative example of flow cytometry analysis with propidium iodide. MDA-MB-231 cells were treated with curcumin and its analogues at 25 μM for 24 h. Numbers in the panels indicate the % of the events in the preG_0_–G_1_ position.

In the two TNBC cell lines, Compounds **1**–**3** showed increased or at least equivalent cell death induction than curcumin. The cells were treated for 24 h with curcumin and its derivatives at concentrations close to the corresponding IC_50_ values. The flow cytometry analysis with propidium iodide revealed that in SUM 149 cells only Compound 1 determined a significant block in a pre-G_0_-G_1_ position, while in MDA-MB-231 cells the order of potency in inducing cell death was Compound **1** > Compound **3** > curcumin (Figure 4 and Figure 5). Anyway, the results appeared to be in agreement with the cytotoxicity data.

#### 2.2.4. NF-κB Inhibition

**Figure 6:**
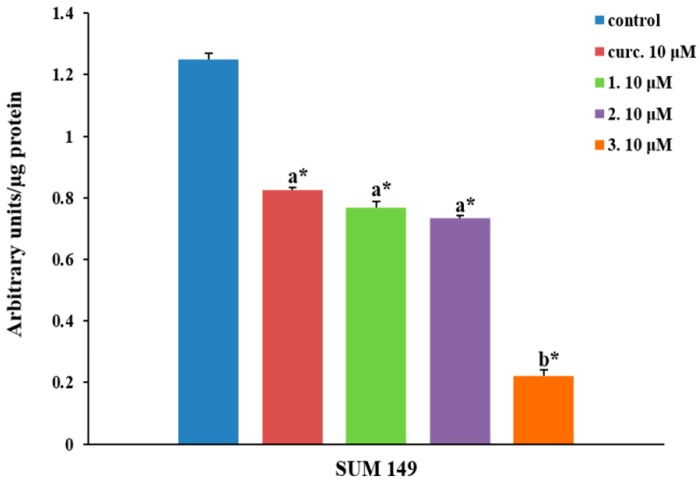
NF-κB (p65 subunit) DNA binding capacity in nuclear extracts of SUM 149 cells. The cells were treated for 24 h with curcumin and derivatives **1**–**3**. Results (mean ± standard error of two experiments carried out in duplicate) are expressed as arbitrary units/μg protein of cells nuclear extracts. Different letters (a and b) in the column of the cell lines and treatments represent significant differences among the different treatments. * Differences when treatments are compared to the control, *p* < 0.01.

**Figure 7:**
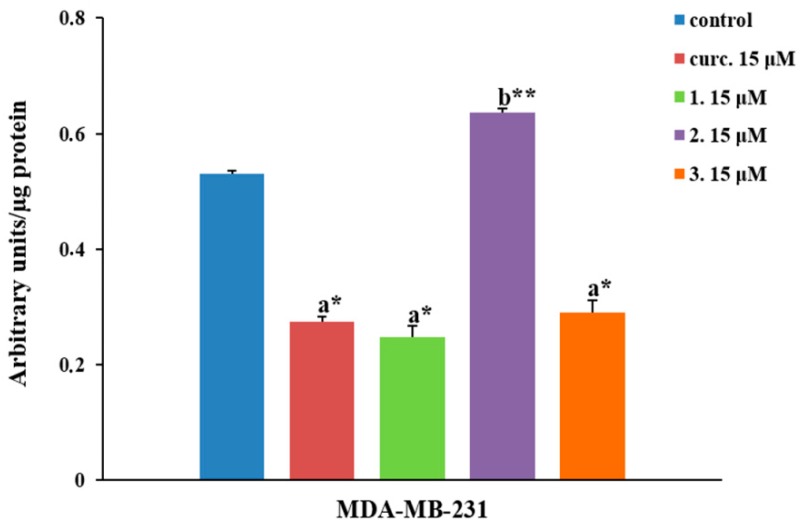
NF-κB (p65 subunit) DNA binding capacity in nuclear extracts of MDA-MB-231 cells. The cells were treated for 8 h with curcumin and derivatives **1**–**3**. Results (mean ± standard error of two experiments carried out in duplicate) are expressed as arbitrary units/μg protein of cells nuclear extracts. Different letters (a and b) in the column of the cell lines and treatments represent significant differences among the different treatments. Differences when treatments are compared to the control: ** *p* < 0.05, * *p* < 0.01.

Since curcumin is an inhibitor of the nuclear activation of NF-κB, we analyzed the capacity of curcumin derivatives **1**–**3** to inhibit NF-κB DNA-binding activity by TransAM assays. Each cell line was treated for 8 h and 24 h with curcumin and its derivatives, at the relative IC_50_ values. In the SUM 149 cell line we observed a high reduction of NF-κB DNA-binding activity for Compounds **1**–**3**, in particular, Compound **3** exhibited a stronger decrease than curcumin, only after 24 h of treatment (Figure 6). In MDA-MB-231 cells Compounds **1** and **3** were NF-κB inhibitors but only Compound **1** showed a greater effect than curcumin, after 8 h of treatment (Figure 7).

**Figure 8:**
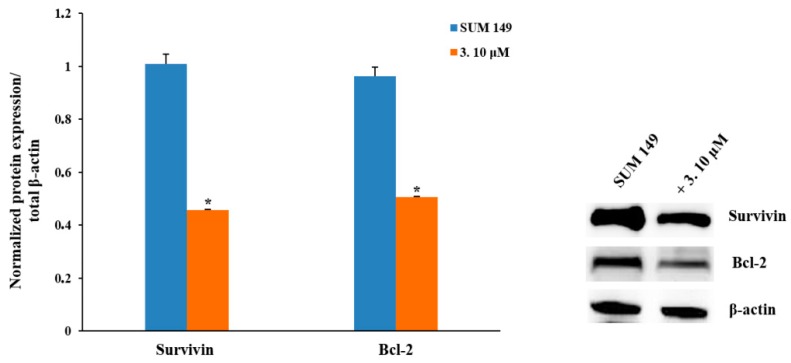
Western blot analysis of the levels of Survivin and Bcl-2 in SUM 149 cells treated for 24 h with Compound **3**. On the left the results are expressed as mean ± standard error (SE) of two different experiments; on the right, the results of a representative experiment. * Differences when treatments are compared to the control, *p* < 0.01.

**Figure 9:**
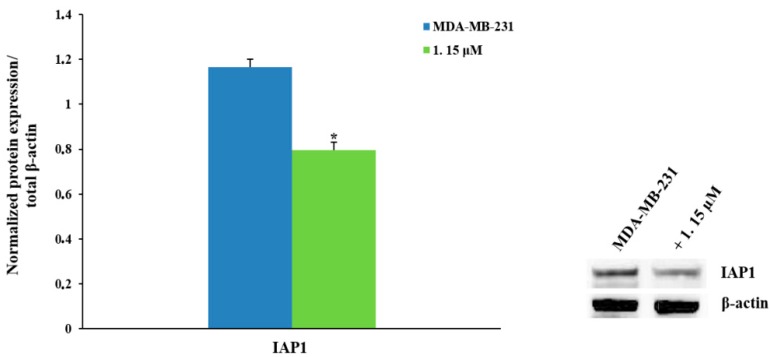
Western blot analysis of the levels of IAP1 in MDA-MB-231 cells treated for 8 h with Compound **1**. On the left are the results expressed as mean ± standard error (SE) of two different experiments; on the right, the results of a representative experiment. * Differences when treatments are compared to the control, *p* < 0.01.

In order to verify if the three analogues alter the expression of some targets of NF-κB, like curcumin, western blot analysis was used. The two cell lines were treated with curcumin and derivatives **1**–**3** in the same conditions. In line with the results of NF-κB DNA-binding activity inhibition, only Compounds **1** and **3** caused a decrease of expression of some targets in the same cell lines. In particular, in SUM 149 cells only Compound **3** caused a decreased expression of Survivin (a 55% of reduction respect to control) and Bcl-2 (a 48% of reduction respect to control), while in MDA-MB-231 cells only Compound 1 caused a reduction of IAP1 expression (a 32% of reduction with respect to the control) (Figure 8 and Figure 9). 

## 3. Conclusions

In summary, the synthesis of three new curcumin derivatives has been described, one of which has been prepared by an original procedure via sulfenic acid condensation. Five curcumin derivatives have been analyzed in a study on TNBC cell lines, of which Compounds **1**–**3** showed cytotoxic and pro-apoptotic activity towards the cellular models studied. Among the three compounds, **1** and **3** seem to be able to inhibit the activation of NF-κB and the expression of some of its targets in a stronger way than curcumin and in a mode which is peculiar with respect to the two TNBC cell lines examined. This difference could be due to some differences, both phenotypic and molecular, between the two cell lines studied. In fact, SUM 149 is a model of inflammatory breast cancer, a basal-like subtype, while MDA-MB-231 is claudin-low subtype. Other authors observe different responses to drugs between these two cell lines though both are TNBC models [44,45].

We thought that SUM 149 and MDA-MB-231 cells could represent the enormous clinical heterogeneity which is found in patients. In fact, although both cell lines belong to the triple negative type, they represent a good study model to compare the response to drugs just for their different characteristics. 

Even though Compound **2** has an IC_50_ inferior than curcumin in both cell lines, on NF-κB it has comparable or lower effects to those of curcumin (respectively in SUM 149 cells and in MDA-MB-231 cells). Its lower efficacy on the inhibition of the targets could depend precisely on the effect on NF-κB that was more limited compared to Analogues **1** and **3**. This result would not seem to depend on the chemical structure of Compound **2**, rather than on the peculiarity of the mechanism of action of the different analogues on the inhibition of the transcription factor. Of all three compounds, Compound **3** appears to be the most promising, given that in both cell lines it shows strong inhibitory effects of cell proliferation and pro-apoptotic as well as a downregulation of NF-κB activity. For these biological effects, this compound appears to be worthy for further analysis, as in vivo assays, to demonstrate the real efficacy as a new anticancer agent. 

Taken together, our data corroborate potentiality of curcumin as a lead compound in cancer diseases. In particular, in this work we highlighted Analogues **1**, **2** and **3** were effective molecules towards triple negative cancer cell lines. Their cytotoxic and pro-apoptotic capacities with ability to interfere with NF-κB pathway can contribute to paving the way to their use as new anticancer agents.

## 4. Materials and Methods

### 4.1. Synthesis

Curcumin from *Curcuma longa* (C1386, purity > 65%, Sigma-Aldrich) was purified by column chromatography on silica gel using CHCl_3_/hexane 90:10 as eluent; the other commercial reagents and solvents were used without further purification. The reactions were monitored by TLC on commercially available precoated plates (silica gel 60 F254) and the products were visualized with vanillin (1 g dissolved in MeOH (60 mL) and conc. H_2_SO_4_ (0.6 mL)) and/or by a UV lamp. Silica gel 60 was used for column chromatography. ^1^H and ^13^C NMR spectra were recorded in CDCl_3_ with a Varian 500 spectrometer (at 500 MHz for ^1^H and 125 MHz for ^13^C). Chemical shifts are given in parts per million (ppm) referenced to the residual protons in CDCl_3_ (δ = 7.27 ppm for ^1^H NMR and δ = 77.0 ppm for ^13^C NMR) solvent. Multiplicities of the signals are abbreviated as follows: s = singlet; bs = broad singlet; d = doublet; t = triplet; and m = multiplet. NMR peak assignments are supported by homonuclear (COSY, Correlation Spectroscopy) and heteronuclear correlation ^1^H-^13^C spectroscopy (HSQCAD). Coupling constants (*J*) are given in Hertz. We have chosen to give the same number at two equivalent carbon atoms of Compounds **3**–**5**, as shown in the structures reported below (equivalent carbons are colored blue). Combustion analyses were carried out on a FISONS EA1108 elemental analyzer.

#### 4.1.1. (1E,6E)-1-(4-hydroxy-3-methoxyphenyl)-7-(3-methoxy-4-propargyloxyphenyl)hepta-1,6-diene -3,5-dione (1) and (1E,6E)-1,7-bis(3-methoxy-4-propargyloxyphenyl)hepta-1,6-diene-3,5-dione (2)

Curcumin (300 mg, 0.81 mmol) was dissolved in 15 mL of dry acetone, then propargyl bromide (61 mg, 0.40 mmol) and K_2_CO_3_ (56 mg, 0.40 mmol) were added. The mixture was stirred under argon at reflux temperature for 24 h. After that, a second addition of propargyl bromide (61 mg, 0.40 mmol) and K_2_CO_3_ (56 mg, 0.40 mmol) was made. The reaction was monitored for another 24 h, using TLC on silica gel (CHCl_3_/hexane 9:1). The solvent was removed under reduced pressure. The residue was suspended in water and extracted with ethyl acetate. The combined organic layers were dried over anhydrous Na_2_SO_4_, filtered and concentrated under reduced pressure. The crude was purified by column chromatography (CHCl_3_/hexane 9:1) to obtain Compound **1** as an orange solid in 47% yield, R*f* 0.6 (CHCl_3_/hexane 9:1). The same column provided **2** as a yellow solid, 34% yield, R*f* 0.8 (CHCl_3_/Hexane 9:1). The ^1^H and ^13^C NMR data are consistent with the one reported in the literature [38].

#### 4.1.2. 4-phenylsulfanyl-(1E,6E)-1,7-bis(4-hydroxy-3-methoxyphenyl)hepta-1,6-diene-3,5-dione (3)

**Figure 10:**
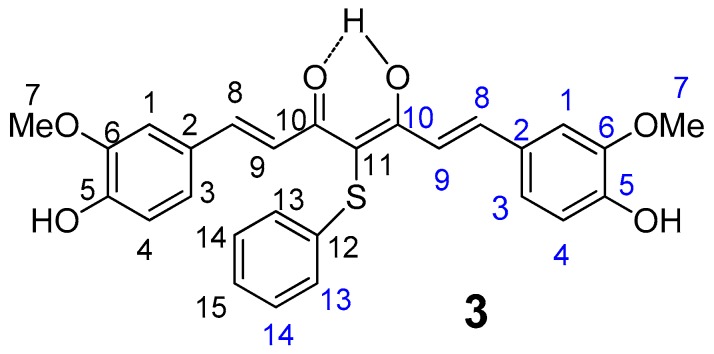
Structure of Compound 3.

**Figure 11:**
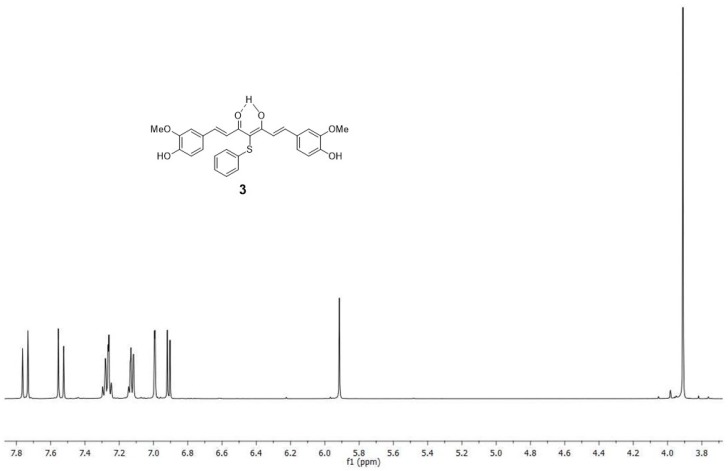
^1^H NMR spectrum of Compound 3 in CDCl_3_ as solvent.

**Figure 12:**
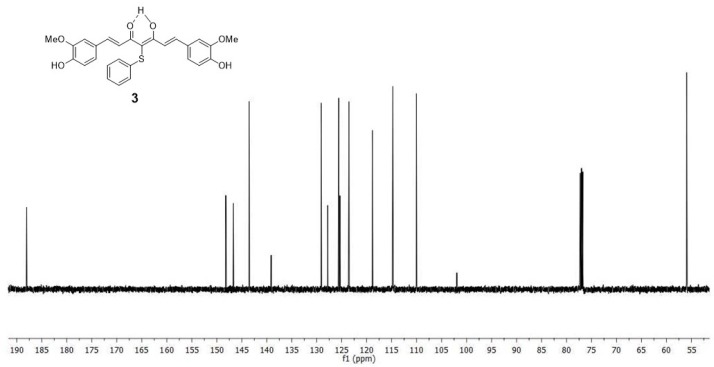
^13^C NMR spectrum of Compound 3 in CDCl_3_ as solvent.

Curcumin (643 mg, 1.77 mmol) and methyl 3-(phenylsulfinyl)propanoate 7 [43] (366 mg, 1.77 mmol) were dissolved in DCE (45 mL) and refluxed (83 °C) for 48 h. The reaction was monitored by TLC (100% DCM), until disappearance of sulfoxide 7. The solvent was removed under reduced pressure and the crude was purified by column chromatography (hexane/DCM 2:8) to obtain Compound **3** (Figure 10) as an orange solid in 65% yield. Rf 0.3 (hexane/DCM 2:8); ^1^H (CDCl_3_): δ 7.73 (2H, d, J = 15.6 Hz, H-8), 7.52 (2H, d, J = 15.6 Hz, H-9), 7.28-7.22 (4H, m, H-13,14), 7.14-7.08 (3H, m, H-3,15), 6.97 (2H, d, J = 1.5 Hz, H-1), 6.89 (2H, d, J = 8.3 Hz, H-4), 5.89 (2H, s, OH), 3.895 (6H, s, H-7); ^13^C NMR (CDCl_3_): δ 188.0 (C-10), 148.2 (Cq), 146.7 (Cq), 143.5 (C-8), 139.1 (Cq), 129.1 and 125.6 (C-13 and C-14), 127.8 (Cq), 125.3 (C-15), 123.5 (C-3), 118.8 (C-9), 114.7 (C-4), 110.0 (C-1), 101.9 (C-11), 55.9 (C-7). Anal. Calcd for C_27_H_24_O_6_S (476,54): C, 68.05; H, 5.08. Found: C, 67.91; H, 5.07. Figure 11 and Figure 12 show respectively ^1^H NMR spectrum and ^13^C NMR spectrum of Compound 3 in CDCl_3_ as solvent.

#### 4.1.3. (1E,6E)-1-(4-hydroxy-3-methoxyphenyl)-7-[3-methoxy-4(4-methylbromobenzyl)oxyphenyl] hepta-1,6-diene-3,5-dione (4) and (1E,6E)-1,7-bis [3-methoxy-4(4-methylbromobenzyl) oxyphenyl]hepta-1,6-diene-3,5-dione (5) 

**Figure 13:**
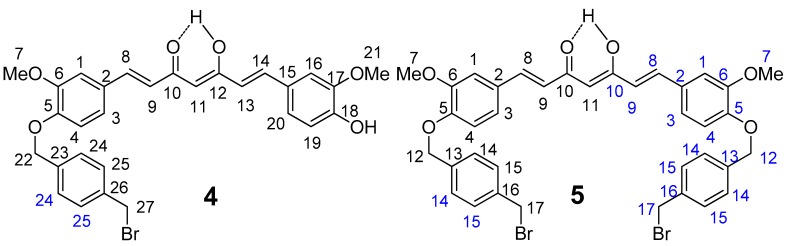
Structure of Compounds 4 and 5.

**Figure 14:**
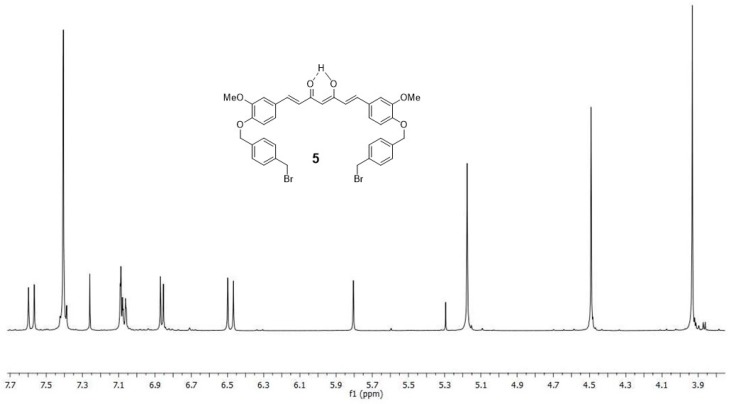
^1^H NMR spectrum of Compound 5 in CDCl_3_ as solvent.

**Figure 15:**
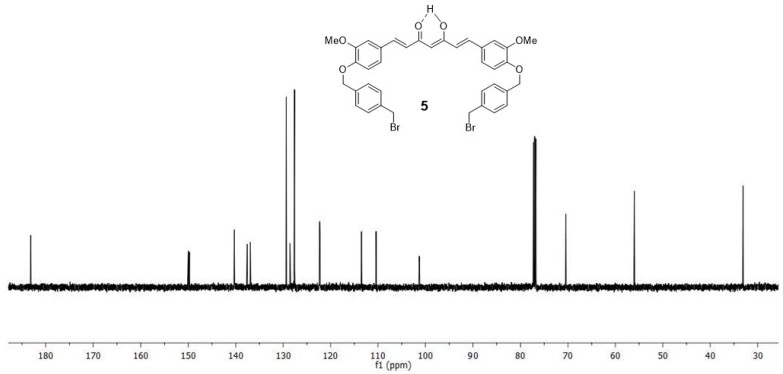
^13^C NMR spectrum of Compound 5 in CDCl_3_ as solvent.

Dibromo-p-xylene 6 (363, 1.37 mmol) was dissolved in a solution of curcumin (1 g, 2.74 mmol) in dry acetone (100 mL), under argon. K_2_CO_3_ (190 mg, 1.37 mmol) was then added and the mixture stirred at reflux for 12 h. After that, a second addition of dibromo-p-xylene 6 (363, 1.37 mmol) and K_2_CO_3_ (190 mg, 1.37 mmol) was made and the reaction was maintained at reflux for 24 h. The reaction was monitored using TLC (hexane/ethyl acetate 1:1). The crude was filtered, and the solvent was removed from the solution under reduced pressure. The residue was suspended in water and extracted with ethyl acetate. The crude was purified by column chromatography (hexane/ethyl acetate 7:3) to obtain Compound **5** (Figure 13) as an orange solid in 40% yield. R*f* 0.7 (hexane/EtOAc 7:3); ^1^H NMR (CDCl_3_): δ 7.58 (2H, d, *J* = 15.6 Hz, H-8), 7.40 (8H, bs, H-14,15), 7.09 (2H, d, *J* = 1.5 Hz, H-1), 7.07 (2H, dd, *J* = 8.3 Hz, *J* = 1.5 Hz, H-3), 6.86 (2H, d, *J* = 8.3 Hz, H-4), 6.48 (2H, d, *J* = 15.6 Hz, H-9), 5.80 (1H, s, H-11), 5.17 (4H, s, H-12), 4.49 (4H, s, H-17), 3.93 (6H, s, H-7); ^13^C NMR (CDCl_3_): δ 183.2 (C-10), 149.9 (Cq), 149.7 (Cq), 140.2 (C-8), 137.5 (Cq), 136.9 (Cq), 129.3 and 127.5 (C-14 and C-15), 128.5 (Cq), 122.3 and 122.2 (C-3 and C-9), 113.4 (C-4), 110.4 (C-1), 101.3 (C-11), 70.4 (C-12), 56.0 (C-7), 33.1 (C-17). Anal. Calcd for C_37_H_34_Br_2_O_6_ (734,47): C, 60.51; H, 4.67; Found: C, 60.70; H, 4.68. Figure 14 and Figure 15 show respectively ^1^H NMR spectrum and ^13^C NMR spectrum of Compound 5 in CDCl_3_ as solvent.

**Figure 16:**
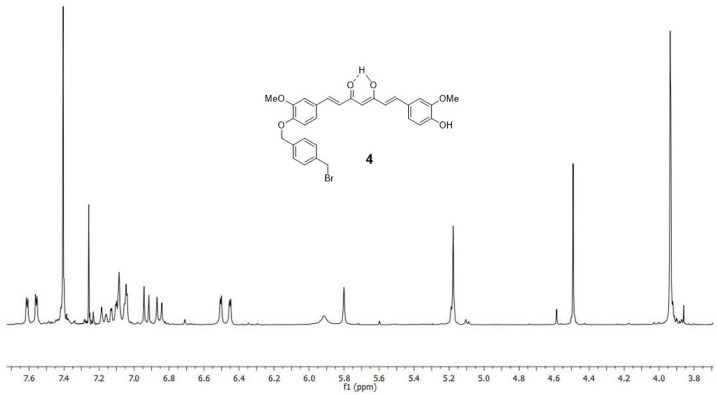
^1^H NMR spectrum of Compound 4 in CDCl_3_ as solvent.

**Figure 17:**
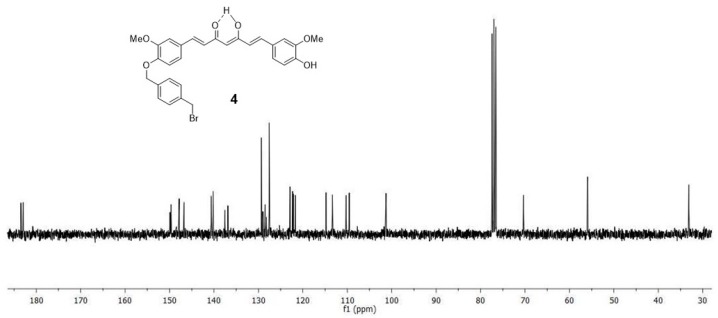
^13^C NMR spectrum of Compound 4 in CDCl_3_ as solvent.

The same column provided Compound **4** (Figure 13) as a yellow solid, 32% yield. R*f* 0.4 (hexane/EtOAc 7:3); ^1^H NMR (CDCl_3_): δ = 7.59 (1H, d, *J* = 15.8 Hz, H-8), 7.58 (1H, d, *J* = 15.8 Hz, H-14), 7.41 (4H, m, H-24,25), 7.14-7.05 (4H, m, H-1,3,16,20), 6.93 and 6.86 (2H, two d, *J* = 8.2 Hz, H-4,19), 6.48 (1H, d, *J* = 15.8 Hz, H-13), 6.47 (1H, d, *J* = 15.8 Hz, H-9), 5.92 (1H, bs, OH), 5.80 (1H, s, H-11), 5.18 (2H, s, H-22), 4.49 (2H, s, H-27), 3.95 and 3.94 (6H, two s, H-7,21); ^13^C NMR (CDCl_3_): δ 183.4 and 182.9 (C-10,12), 149.8 (Cq), 149.6 (Cq), 147.8 (Cq), 146.7 (Cq), 140.6 and 140.2 (C-8,14), 137.5 (Cq), 136.8 (Cq), 129.3 and 127.5 (C-24,25), 128.9 (Cq), 128.4 (Cq), 122.8, 122.3, 122.1 and 121.6 (C-3,9,13,20), 114.7 and 113.3 (C-4,19), 110.2 and 109.5 (C1 and C16), 101.2 (C-11), 70.3 (C-22), 56.0 and 55.9 (C-7,21), 33.1 (C-27). Anal. Calcd for C_29_H_27_BrO_6_ (551,43): C, 63.17; H, 4.94; Found: C, 63.33; H, 4.95. Figure 16 and Figure 17 show respectively ^1^H NMR spectrum and ^13^C NMR spectrum of Compound 4 in CDCl_3_ as solvent.

### 4.2. Cell Lines

Dr. Elda Tagliabue (Molecular Targeting Unit, Department of Experimental Oncology and Molecular Medicine, Fondazione Institute of Hospitalization and Scientific Care, National Cancer Institute, Milan, Italy) kindly provided us the human breast cancer cell lines: MDA-MB-231(ATCC: HTB-26—Rockville, MD, USA) and SUM 149 (SUM149PT—Asterand Bioscience Detroit, MI). The first was cultured in RPMI-1640 and the second was cultured in DMEM/F-12 supplemented with insulin (5 μg/mL). The cells were authenticated using the short tandem repeat profiling method in their Institute. Prof. Giulio Ghersi (STEBICEF Department, University of Palermo, Italy) kindly provided us the non-tumorigenic cell line 1-7HB2 (ECACC 10081201—Cancer Research Technology, London, UK) that was cultured in DMEM low glucose supplemented with hydrocortisone (5 μg/mL) and insulin (10 μg/mL). HL60, obtained from ATCC^®^ (CCL-240, Rockville, MD, USA), and its variant HL60R, obtained by exposure to gradually increasing concentrations of doxorubicin, were cultured in RPMI-1640. All media were supplemented with 10% heat-inactivated fetal calf serum, 2 mM L-glutamine, 100 U/mL penicillin and 100 μg/mL streptomycin (all reagents were from EuroClone S.p.A., Milan, Italy; GE Healthcare Life Sciences, Logan, UT, USA). All cell lines were cultured in a humidified atmosphere at 37 °C in 5% CO_2_. 

Cells with a narrow range of passage number (4 ± 6) were tested for Mycoplasma contamination and used for all experiments. After obtaining the cells, the first passage carried out was assigned passage number 1 [35]. 

### 4.3. Cell Growth Aassays

The cells were seeded at 2 × 10^4^ cells/well onto 96-well plates and incubated at 37 °C overnight; at time 0, the medium was replaced with fresh complete medium supplemented of compounds at the indicated concentrations. Following 72 h of treatment, 16 μL of a commercial solution obtained from Promega Corporation (Madison, WI, USA) containing 3-(4,5-dimethylthiazol- 2-yl)-5-(3-carboxy methoxyphenyl)-2-(4-sulphophenyl)-2H-tetrazolium (MTS) and phenazine ethosulfate were added. After a incubation in a humidified atmosphere at 37 °C in 5% CO_2_, the bioreduction of MTS dye was evaluated by measuring the absorbance of each well at 490 nm, using a microplate absorbance reader (iMark Microplate Reader; Bio-Rad Laboratories, Inc., Hercules, CA, USA). Cell growth inhibition was expressed as a percentage (mean ± SE) of the absorbance of the control cells [35]. 

### 4.4. Anti- and Pro-oxidant Activity

The antioxidant activity was evaluated by the DPPH stable radical method. Trolox (6-hydroxy-2,5,7,8-tetramethyl-chroman-2-carboxylic acid) curve was used as the positive control. 100 μL of sample was added to aliquots of a solution made up with DPPH (4.8 mg) in MeOH (200 mL), and the mixture was incubated in the dark, for 1 h at room temperature. The absorbance was measured using a UV-VIS spectrophotometer, at 517 nm. The results were plotted as the percentage of absorbance disappearance at 517 nm [(1-A/A_0_) × 100] against the amount of sample divided by the initial concentration of DPPH; lower absorbance values of reaction mixture indicate higher free radical scavenging activity. ED_50_ corresponds to micrograms of fraction able to consume half the amount of free radical divided by micromoles of initial DPPH. The results were expressed as antiradical capacity (ARC), which is the inverse of ED_50_. Each point was acquired in triplicate.

Pro-oxidant effects were examined by cell counting, adding N-acetyl-L-cysteine (NAC), an antioxidant molecule, 1h before compounds. Results were expressed as mean ± standard error (SE) of at least three different experiments performed in duplicate. All the chemicals were supplied by Sigma Aldrich Srl, Milan, Italy [46]. 

### 4.5. Evaluation of Cell Death by Flow Cytometry 

Cells were washed twice with ice-cold PBS and then resuspended in a hypotonic fluorochrome solution containing propidium iodide (PI) 50 μg/mL in 0.1% sodium citrate plus 0.03% (v/v) Nonidet P-40, at 1×10^6^/^mL^. After incubation (1 h) in this solution, the samples were filtered through nylon cloth, 40 μm mesh, and their fluorescence was analyzed using a FACSCanto instrument (Becton Dickinson, Montain View, CA, USA). The data were analyzed with BD FACSDiva software v.6.1.2. (Becton Dickinson). Cell death was determined by evaluating the percentage of events accumulated in the preG_0_-G_1_ position [13].

### 4.6. NF-κB Activation

The DNA-binding capacity of NF-κB (p65 subunit) was defined in the nuclear extracts of SUM 149 and MDA-MB-231 cells using the TransAM NF-κB and Nuclear Extract kits (Active Motif, Carlsbad, CA, USA). The determination of binding capacity was based on a 96-well plate, on which an oligonucleotide containing the NF-κB consensus binding site was fixed. By use of an antibody directed against an epitope on p65, it may revealed NF-κB bound to the oligonucleotide. After addition of a horseradish peroxidase-conjugated secondary antibody, a sensitive colorimetric readout was quantified by densitometry (iMark Microplate Reader; Bio-Rad Laboratories, Inc.). The control of specificity of the assay carried out according to the indications of manufacturer’s protocol. The results were expressed as arbitrary units: one unit indicated the DNA binding capacity exerted by 2.5 μg whole cell extract from Jurkat cells/microgram of protein from the nuclear extracts. Jurkat cells, stimulated with 12-O-tetradecanoylphorbol-13-acetate and calcium ionophore, are the positive control for NF-κB p65 activation [35].

### 4.7. Western Blotting

Whole-cell lysates were obtained from breast cancer cells using RIPA buffer (Santa Cruz Biotechnology Inc., Dallas, TX, USA) and 25 μg protein was subjected to 10% SDS-PAGE and transferred to Hybond-P membranes (GE Healthcare Europe GmbH, Freiburg, Germany). Filters were incubated with primary antibodies raised against β-actin (Sigma-Aldrich Srl, Milan, Italy), Bcl-2 (Santa Cruz Biotechnology Inc., Dallas, TX, USA), XIAP (Cell Signaling Technology, Danvers, MA), Survivin (Novus Biologicals, Littleton, CO), IAP1 (Cell Signaling Technology, Danvers, MA). An enhanced chemiluminescence detection kit (SuperSignal West Femto Maximum Sensitivity Substrate, Thermo Scientific) and the Versa DOC imaging system (BioRad) were used to visualized hybridization.

Immunoblots were quantified by densitometry, the data were expressed as arbitrary units (protein/β-actin) [35].

### 4.8. Statistical Analysis

Results are given as means ± standard error (SE). Statistical analysis was performed by analysis of variance (one-way ANOVA) followed by Tukey’s test. The software used is Statistica ver. 12 (StatSoft Inc. 1984–2014).

## Figures and Tables

**Table 1:** Cell growth inhibitory effects of Compounds **1**–**5** and curcumin evaluated after 72 h of treatment by MTS assays.

Compounds	SUM149 IC_50_ (μM)	MDA-MB-231 IC_50_ (μM)	1-7HB2 IC_50_ (μM)
Curcumin (curc) 1	14.0 ± 0.29 11.2 ± 1.30	25.5 ± 0.35 18.0 ± 0.41	37.5 ± 0.51 20.0 ± 0.22
2	13.2 ± 1.59	20.0 ± 0.00	not reached
3	13.5 ± 0.88	15.0 ± 0.85	12.5 ± 0.43
4	not reached	not reached	not reached
5	not reached	not reached	not reached

Data are expressed as the concentrations which inhibit 50% (IC_50_) cell growth and are means ± SE of at least three separate experiments.

**Table 2:** Cell counting analysis in SUM 149 cells (**A**) and in MDA-MB-231 cells (**B**) after treatment with antioxidant NAC at 2mM before exposure to the curcumin and its analogues at the IC_50_ values. Data are expressed as mean ± standard error (SE).

**A**	**Cell Lines and Treatments**	**Cell Viability (%)**
	*SUM 149*	
	+ NAC 2 mM ^a,^*	100.0 ± 9.5
	+ curc. 14 μM ^bc,^* + 1. 11 μM ^bc,^*	32.0 ± 2.1 28.0 ± 3.2
	+ 2. 13 μM ^bd,^*	36.6 ± 1.8
	+ 3. 14 μM ^bc,^*	34.0 ± 2.1
	+ NAC + curc ^de,^*	74.5 ± 0.0
	+ NAC + 1 ^ae,^*	100.0 ± 20.5
	+ NAC + 2 ^ae,^*	74.4 ± 1.4
	+ NAC + 3 ^c,^*	14.0 ± 4.6
**B**	*MDA-MB-231*	
	+ NAC 2 mM ^a,^*	87.0 ± 3.9
	+ curc. 25μM ^ab,^* + 1. 18μM ^b,^*	45.0 ± 4.6 23.0 ± 1.4
	+ 2. 20μM ^ab,^*	44.0 ± 4.9
	+ 3. 15μM ^b,^*	25.6 ± 0.7
	+ NAC + curc ^abc,^*	60.0 ± 2.1
	+ NAC + 1 ^ab,^*	53.5 ± 0.7
	+ NAC + 2 ^ac,^*	78.0 ± 1.8

Significant differences among the treatments of each cell line are represented by different letters (a, b, c, d and e) in the column (Cell lines and treatments). * Differences when treatments are compared to the control, *p* < 0.05.

**Table 3:** DPPH free radical scavenging activity. The results were expressed as the antiradical capacity (ARC), which is the inverse of the ED_50_.

	ED_50_	ARC (1/ED_50_)
Trolox	12.0 µM	5
Curcumin 1	7.5 µM >100 µM	8 -
2	>100 µM	-
3	19.2 µM	3.1
